# Polyunsaturated ω-3 fatty acids inhibit ACE2-controlled SARS-CoV-2 binding and cellular entry

**DOI:** 10.1038/s41598-021-84850-1

**Published:** 2021-03-04

**Authors:** Anna Goc, Aleksandra Niedzwiecki, Matthias Rath

**Affiliations:** grid.418580.0 Dr. Rath Research Institute BV, 5941 Optical Ct., San Jose, CA 95138 USA

**Keywords:** Microbiology, Virology, SARS-CoV-2

## Abstract

The strain SARS-CoV-2, newly emerged in late 2019, has been identified as the cause of COVID-19 and the pandemic declared by WHO in early 2020. Although lipids have been shown to possess antiviral efficacy, little is currently known about lipid compounds with anti-SARS-CoV-2 binding and entry properties. To address this issue, we screened, overall, 17 polyunsaturated fatty acids, monounsaturated fatty acids and saturated fatty acids, as wells as lipid-soluble vitamins. In performing target-based ligand screening utilizing the RBD-SARS-CoV-2 sequence, we observed that polyunsaturated fatty acids most effectively interfere with binding to hACE2, the receptor for SARS-CoV-2. Using a spike protein pseudo-virus, we also found that linolenic acid and eicosapentaenoic acid significantly block the entry of SARS-CoV-2. In addition, eicosapentaenoic acid showed higher efficacy than linolenic acid in reducing activity of TMPRSS2 and cathepsin L proteases, but neither of the fatty acids affected their expression at the protein level. Also, neither reduction of hACE2 activity nor binding to the hACE2 receptor upon treatment with these two fatty acids was observed. Although further in vivo experiments are warranted to validate the current findings, our study provides a new insight into the role of lipids as antiviral compounds against the SARS-CoV-2 strain.

## Introduction

Coronaviruses (CoVs) belong to the *Coronaviridae* family of the order *Nidovirales*, which are divided into four genera (α, β, γ, and δ). SARS-CoV-2 strain (also reported as 2019-nCov, 2019-CoV-2, nCoV-2019), which has been identified as a cause of the outbreak of pneumonia in Wuhan, China, in 2019, is classified to the β genus. This novel coronavirus 2019-nCoV has been isolated from human lung (airway) epithelial cells, and showed similarity to the other coronaviruses causing earlier pandemics: the Severe Acute Respiratory syndrome (SARS) in 2002–2004, and the Middle East Respiratory Syndrome (MERS) in 2012^1–3^.

In general, SARS-CoV-2 contains a positive, single-stranded, genomic RNA enveloped with different structural proteins such as spike (S) protein, envelope (E) protein, membrane (M) protein, and the nucleocapsid (N) protein^4–6^. It infects various vertebrates, including humans, causing predominantly respiratory-tract infections, though with diverse clinical manifestations. Recent developments have also revealed that SARS-CoV-2 invades human cells through binding of its surface spike protein to the angiotensin-converting enzyme 2 (ACE2), as its host cognate receptor, present on the membrane of various human cells. This viral-host attachment triggers cell-membrane fusion and subsequently allows virus entry^7–12^. Spike protein of SARS-CoV-2 shares about 76% and 97% of amino acid homology with SARS-CoV and bat coronavirus RaTG13, respectively, while the amino acid sequence of receptor-binding domain (RBD) of SARSCoV-2 is about 74% and 90.1% respectively, homologous to SARS-CoV and RaTG13^8,12^.

Spike protein (S glycoprotein) is a surface-exposed transmembrane molecule consisting of two subunits, S1 and S2, mediating attachment and membrane fusion, respectively. Attachment between the virus and host cells is made possible by the binding of the N-terminal domain (NTD) of the S1 subunit of viral spike protein, which contains the receptor-binding domain, to the human cellular ACE2 receptor. Once the S1 subunit binds to the host sell receptors, membrane fusion is induced when heptad repeat (HR) regions within the S2 subunit undergo a conformational change into an intra-hairpin-helical structure with six helix packet^13–15^. Once this conformational change is complete, the fusion peptide is secured to the membrane of the host cell, allowing the virus to draw closer and to deliver the nucleocapsid protein into the cell. Thus, spike protein and consequently viral binding to the host receptor is the major target in the search for effective therapeutics that might prevent a virus from infecting host cells, and subsequently prove effective against SARS-CoV-2-caused infection^16^.

CoVs spike proteins are class I of viral fusion proteins, and their priming by protease cleavage is required for the initiation of the binding to the receptor, fusion, and viral endocytosis^13^. Based on the recent studies, a two-step consecutive protease cleavage process for “activation” of spike proteins of SARS-CoV-2 seems to be necessary, i.e., cleavage between S1 and S2 and cleavage on S2 subunit itself^17–19^. Depending on CoVs strains and cell types, spike protein may be cleaved by one or several host proteases, such as furin, trypsin, cathepsins, transmembrane protease serine protease-2 (TMPRSS-2), transmembrane protease serine protease-4 (TMPRSS-4), or human airway trypsin-like protease (HAT)^7,20–24^. In the case of SARS-CoV-2, most studies suggest crucial involvement of transmembrane protease serine protease-2 (TMPRSS-2) and cathepsin L as the proteases on target cells determining viral binding and cellular entry^7,8,24,25^. However, other proteases that could promote SARS-CoV-2 entry cannot be excluded.

Lipids are a group of diverse bioactive nutrient and non-nutrient compounds of plant, animal or petrochemical origin, affecting various physiological and biochemical processes with demonstrated impact on human health^26–29^. Commonly defined as hydrophobic, they comprise fatty acids (FA), also known as carboxylic acids, with a short, medium or long acid chain, and include unsaturated oils (liquid at room temperature) and saturated fats (solid at room temperature). Saturated or unsaturated, FAs are the essential building blocks of other structurally complex lipids and lipid containing molecules. They are important dietary sources of energy in animal metabolism, and a number of them have been shown to inactivate enveloped viruses^27,28^. Thus, it is rational to study the specific or general antiviral activity of organic oils and FAs, especially since lipid compounds have not yet been extensively evaluated as anti-SARS-CoV-2 agents, although recently Toelzer et al. reported on a linoleic acid binding pocket in the structure of SARS-CoV-2 spike protein^30^.

The objective of our study was to investigate the potential of selected FAs and lipid-soluble vitamins to inhibit the binding of SARS-CoV-2 to the human ACE2 receptor, and its cellular entry. Using standard and recently developed methodologies, we were able to show that, among 17 tested lipids, polyunsaturated fatty acids (PUFAs) revealed the highest inhibitory binding efficacy of viral RBD of spike protein to the hACE2 receptor. Among them, linolenic acid, eicosapentaenoic acid (EPA), and linoleic acid had the highest binding affinity to spike protein. Moreover, concurrent experiment with SARS-CoV-2 pseudo-virus particles revealed that linolenic acid and EPA could also inhibit their viral binding, membrane fusion, and entry, with the linolenic acid being, however, more cytotoxic than EPA. In addition, we discovered that EPA and, to a lesser extent, linolenic acid, inhibit TMPRSS2 protease and cathepsin L activity, which facilitate the attachment and entry of SARS-CoV-2, but did not affect their expression at the protein level. We also observed that neither of the FAs bound to or affected the activity of the hACE2. In conclusion, this study for the first time documents the anti-SARS-CoV-2 properties of FAs, although further in vivo study is warranted to substantiate these findings.

## Results

### Evaluation of inhibitory properties of FAs and lipid-soluble vitamins on binding of the RBD sequence of SARS-CoV-2 spike protein to the hACE2 receptor

**Figure 1:**
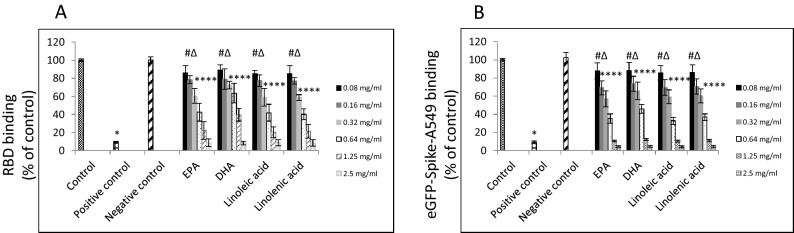
Effects of PUFAs on binding of RBD sequence of SARS-CoV-2 to human ACE2 receptor. (**A**) Binding of RBD sequence of SARS-CoV-2 spike protein to immobilized hACE2 receptor. HRP-conjugated RBD sequence was treated with indicated FAs at different concentrations for 30 min. followed by incubation for 15 min. with hACE2 receptors immobilized on plate. HRP signal was measured at 450 nm. (**B)** Binding of A549 cells expressing SARS-CoV-2 eGFP-spike protein in the present of selected FAs at different concentrations to soluble hACE2 receptor. Binding was performed on plates with immobilized human monoclonal antibody against hACE2 receptor at 10 µg/ml concentration and detected as green fluorescence signal. Data are presented as % of control ± SD; #*p* ≤ 0.05, ∆*p* ≤ 0.01, *p ≤ 0.001. Control—0.025% DMSO, positive and negative controls were provided by the manufacturer.

**Figure 2:**
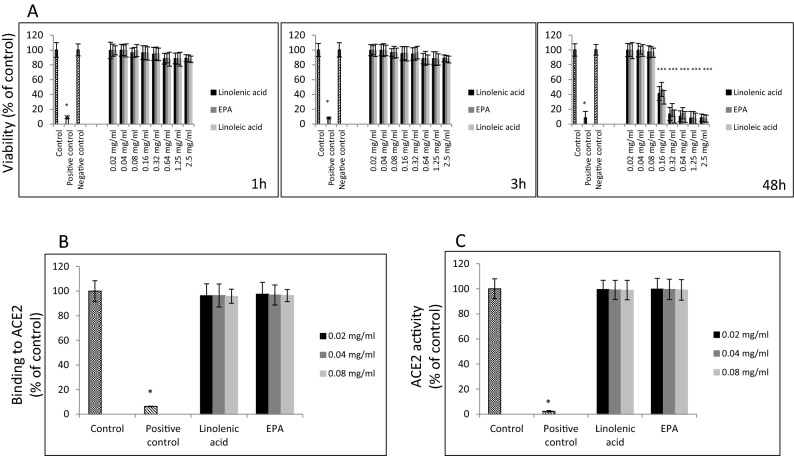
Effects of linolenic acid and EPA on viability of ACE2 expressing A549 cells and on human ACE2 receptor. (**A**) Viability of A549/hACE2 cells after 1 h, 3 h, and 48 h incubation with linolenic acid and EPA at different concentrations using MTT assay. Cell viability is expressed as change in absorbance at 570 nm in % compared to lipid-free control ± SD; positive control—100% dead cells, negative control—addition-free sample. (**B**) Binding of linolenic acid and EPA at indicated concentrations to hACE2 receptor (1.0 μg/ml) immobilized on the plate using human primary anti-ACE2 antibody at 1:500 dilution and HRP-conjugated secondary antibody at 1:1000 dilution, and measuring chemiluminescence signal. Data are presented as % of lipid-free control ± SD; control—0.025% DMSO, positive control—50% DMSO. (**C)** Activity of hACE2 upon treatment with selected FAs and indicated concentrations. Purified hACE2 enzyme at 0.1 ng/µl was incubated with linoleic acid and EPA at different concentrations for 1 h at RT followed by addition of 25 µl fluorogenic substrate for 30 min. Fluorescence signal was measured at Ex/Em = 535/595 nm using spectrofluorimeter. Data are presented as % of lipid-free control ± SD; *p ≤ 0.001. Control—0.025% DMSO, positive control—10% DMSO.

We examined the capability of several types of FAs and lipid-soluble vitamins to inhibit binding of the RBD sequence of the SARS-CoV-2 spike protein to hACE2 receptor. As shown in Table 1, polyunsaturated fatty acids (PUFAs) and vitamin A revealed the utmost binding inhibition at 2.5 mg/ml concentration reaching 100% when a commercially available assay kit was used. Notably, out of four PUFAs assayed, linolenic acid and linoleic acid showed the highest dose-dependent inhibitory effect with 18% inhibition observed at 0.08 mg/ml, as shown in Fig. 1. Accordingly, we examined binding of A549 cells expressing the eGFP-SARS-CoV-2 spike protein pre-incubated for 1 h with these four PUFAs and exposed for 1 h exposure to a soluble hACE2 receptor. Consistently, all four PUFAs blocked binding to the hACE2 receptor in a dose-dependent manner, with 15% inhibition observed at 0.08 mg/ml. Viability study results revealed that tested PUFAs are non-cytotoxic up to 2.5 mg/ml concentration when incubated for 1 h and 3 h or up to 0.08 mg/ml when incubated for 48 h. However, both linolenic acid and linoleic acid seem to be more cytotoxic that EPA or docosahexaenoic acid (DHA). Also, none of these compounds had significant effect on binding to hACE2 receptor itself, nor affected activity of the hACE2 receptor (Fig. 2).

**Table 1:** Effect of FAs and lipid-soluble vitamins on binding with SARS-CoV-2 spike RBD and hACE2 receptor.

Tested fatty acids (2.5 mg/ml)	Binding with RBD^a^ (% of control ± SD)	Binging with ACE2^b^ (% of control ± SD)
**Polyunsaturated**		
Linolenic acid	100 ± 0.02*	4.1 ± 1.2
Eicosapentaenoic acid	100 ± 0.01*	5.2 ± 1.7
Docosahexaenoic acid	100 ± 0.02*	5.3 ± 1.1
Linoleic acid	100 ± 0.01*	4.1 ± 1.1
**Monounsaturated**		
Undecenoic acid	12.5 ± 3.6	7.2 ± 0.9
Palmitoleic acid	10.4 ± 3.6	6.3 ± 1.2
Oleic acid	91.6 ± 5.9*	6.8 ± 1.1
Erucic acid	36.6 ± 2.7^#^	7.5 ± 1.4
Petroselinic acid	88.7 ± 3.1*	5.4 ± 0.8
**Saturated**		
Caprylic acid	20.8 ± 2.6	5.8 ± 0.2
Myristic acid	15.4 ± 1.9	4.5 ± 0.3
Palmitic acid	15.8 ± 1.4	6.7 ± 0.5
Stearic acid	10.7 ± 2.4	5.9 ± 0.8
Arachidic Acid	11.9 ± 2.2	6.2 ± 1.1
**Lipid-soluble vitamins**		
Vitamin D3 (1,25-dihydroxycholecalciferol)	13.3 ± 1.7	4.3 ± 1.4
Vitamin E (alpha-tocopherol)	16.5 ± 2.1	5.8 ± 1.5
Vitamin A (retinol)	100 ± 0.02*	5.9 ± 1.1

Bold indicates the highest binding efficacy.

^a^2.5 mg/ml of tested lipids and lipid-soluble vitamins were first incubated with HRP-conjugated RBD for 30 min at 37 °C and then transferred into a 96-well plate with immobilized hACE2 receptor and incubated for an additional 15 min at 37 °C. Plates were then washed four times and developed with TMB substrate solution for up to 5 min, followed by the addition of stop buffer. Optical density was immediately measured at 450 nm.

^b^2.5 mg/ml of tested lipids and lipid-soluble vitamins were first incubated with hACE2 immobilized on a 96-well plate for 30 min at 37 °C and then hACE2-immobilized plates were washed four times, and incubated with HRP-conjugated RBD for 15 min at 37 °C. Plates were then washed four times and developed with TMB substrate solution for up to 5 min, followed by the addition of stop buffer. Optical density was immediately measured at 450 nm.

^#^*p* ≤ 0.05, *p ≤ 0.001.

### Effect of linolenic acid and eicosapentaenoic acid on binding and entry of SARS-CoV-2 pseudo-virus

**Figure 3:**
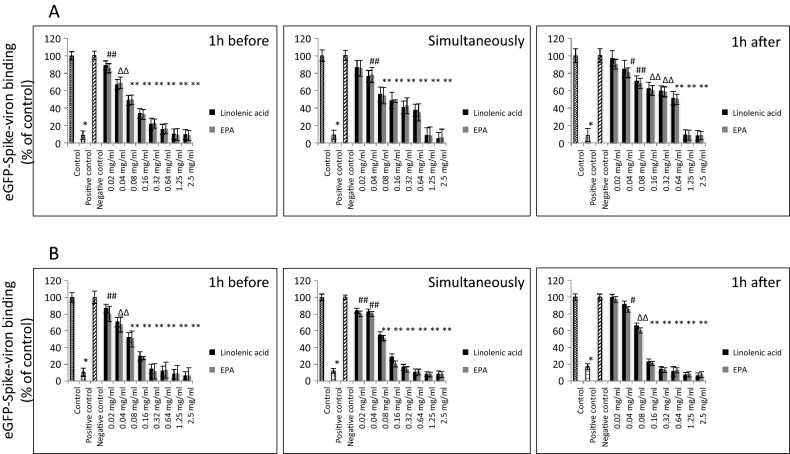
Effects of linolenic acid and EPA on SARS-CoV-2 pseudo-virion binding to human ACE2 receptor. Binding SARS-CoV-2 spike protein encapsulated pseudo-virions to A549 cells stably overexpressing human ACE2 receptor was evaluated with monoclonal antibody. (**A**) Spike-pseudo-virions were treated with indicated FAs at different concentrations for 1 h before inoculation, added simultaneously or 1 h after inoculation of the virions into hACE2/A549 cells. Next, wells were incubated for 1 h at 37^O^C, washed and binding was evaluated by adding primary anti-spike protein monoclonal antibody at 1:1000 dilution followed by secondary HRP-conjugated antibody at 1:2500 dilution and signal measurement at 450 nm. (**B**) Spike-pseudo-virions were treated with indicated FAs at different concentrations for 1 h before inoculation, added simultaneously or 1 h after inoculation of the virions into hACE2/A549 cells. Next, wells were incubated for 3 h at 37^O^C, washed and binding was evaluated by adding primary anti-spike protein monoclonal antibody at 1:1000 dilution followed by secondary HRP-conjugated antibody at 1:2500 dilution and signal measurement at 450 nm. Data are presented as % of control ± SD; #*p* ≤ 0.05, ∆*p* ≤ 0.01, *p ≤ 0.001. Controls—0.025% DMSO, positive and negative controls were provided by the manufacturer.

Subsequently we determined whether SARS-CoV-2 spike protein pseudo-virions binding and entry to A549/hACE2 cells could also be blocked by selected PUFAs. Considering inhibitory binding efficacy and cytotoxicity, we selected linolenic acid and EPA for our further study. Both linolenic acid and EPA, when pre-incubated with pseudo-virus for 1 h, added simultaneously with pseudo-virus or added 1 h after exposure to pseudo-virus, resulted in blocking the virions binding to A549/hACE2 cells from 15 to 100%, regardless of pseudo-viral exposure time, although, a 3-h incubation period seems to show increased binding inhibitory effects by both tested PUFAs (Fig. 3).

**Figure 4:**
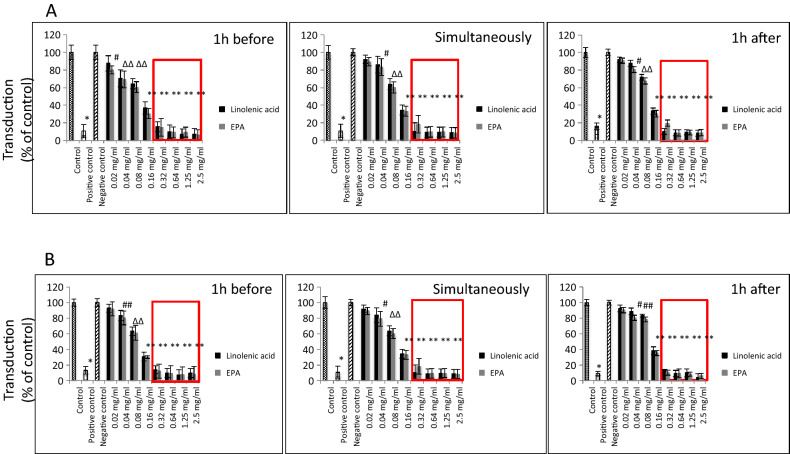
SARS-CoV-2 eGFP-luciferase-pseudo-virion binding and cellular entry. Binding and entry of SARS-CoV-2 pseudo-virions with encapsulated eGFP-luciferase spike protein was evaluated without spinfaction and with spinfaction. (**A**) A549 cells stably overexpressing hACE2 receptor were inoculated (no spinfection) with SARS-CoV-2 pseudo-virions treated with indicated FAs at different concentrations and time. After 48 h post-inoculation time, transduction efficacy was measured according to luciferase activity. (**B**) Binding and entry of SARS-CoV-2 pseudo-virions with encapsulated eGFP-luciferase Spike protein. A549 cells stably overexpressing hACE2 receptor were inoculated (spinfection) with SARS-CoV-2 pseudo-virions treated with indicated FAs at different concentrations and time. After 48 h post-inoculation time, transduction efficacy was measured according to luciferase activity. Data are presented as % of control ± SD; #*p* ≤ 0.05, ∆*p* ≤ 0.01, *p ≤ 0.001. Control—0.025% DMSO, positive control—bald SARS-CoV-2 eGFP-luciferase-pseudo-virions, negative control—ΔG-luciferase rVSV pseudo-typed particles; red fame—concentrations that showed 85–100% cytotoxicity.

**Figure 5:**
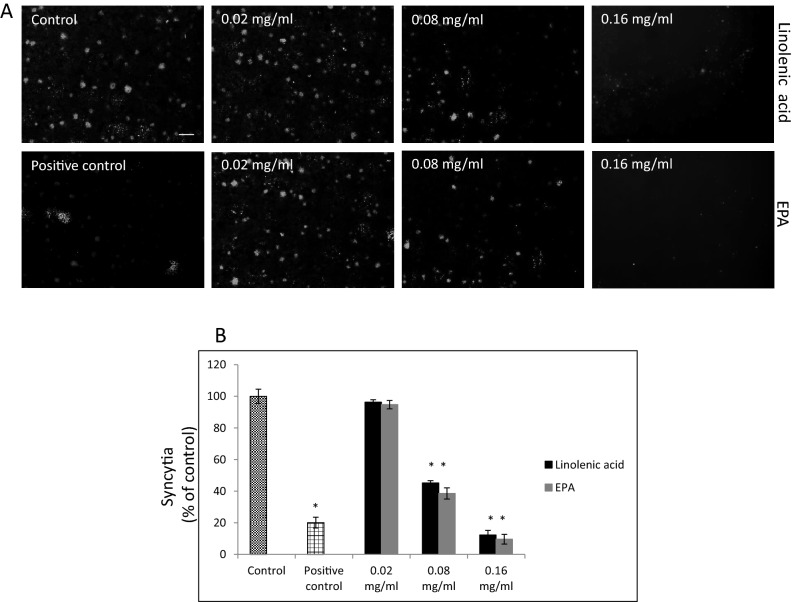
Effect of selected FAs on fusion to human ACE2 receptor overexpressing A549 cells. (**A**). Cell–cell fusion of A549 cells expressing eGFP spike protein with A549 cells stably expressing human ACE2 receptor. A549 cells expressing eGFP spike protein were pre-treated with indicated FAs at different concentrations for 1 h at 37 °C and co-cultured for additional 4 h at 37 °C with A549 cells stable expressing human ACE2 receptor. The scale bar indicates 250 µm. (**B**) Quantitative analysis of formed syncytia. Experiments were done in triplicate and repeated three times. Data are presented as % of control ± SD; *p ≤ 0.001. Control—0.025% DMSO, positive control—20 μg/ml anti-ACE2 antibody.

By using SARS-CoV-2 eGFP-luciferase spike protein pseudo-virions we also evaluated the effect of linolenic acid and EPA on viral entry into A549 cells stably overexpressing hACE2 receptor with spinfection and without spinfection. We used spinfection to check if differences will be seen when the attachment of the spike proteins to ACE2 receptors is prompted by mechanical force as opposed to virions that are allowed freely to attach to ACE2 receptors. Introduction of spinfection revealed that there is a still inhibition taking place pointing to yet other factors that could be involved in binding of viral particles to ACE2 molecule. As shown in Fig. 4, A549/hACE2 cells gave about 20% to 60%, dose-dependent decrease in luciferase activity, when virions were either pre-incubated for 1 h or added simultaneously with the PUFAs. Similar effect was achieved when the PUFAs were added 1 h after cells’ exposure with pseudo-typed virions. In the latter case, the binding blockage to A549/hACE2 cells ranged from 20 to 40% after 48 h post pseudo-viral exposure at non-toxic concentrations (Fig. 4). Inhibition observed at higher than 0.32 mg/ml concentrations of linolenic acid and EPA relates to cell toxicity effects (indicated on Fig. 4 by large rectangles). Additionally, fusion of A549/eGFP-spike cells to A549/hACE2 cells was inhibited by about 60% to 90% in the presence of linolenic acid and EPA, respectively, as presented in Fig. 5, showing representative fields of SARS-CoV-2 spike protein expressing cells attachment. Both PUFAs consistently blocked cell–cell, fusion although linolenic acid showed a lesser inhibition compared with EPA.

### Effect of linolenic acid and eicosapentaenoic acid on host cellular proteases TMPRSS2 and cathepsin L

**Figure 6:**
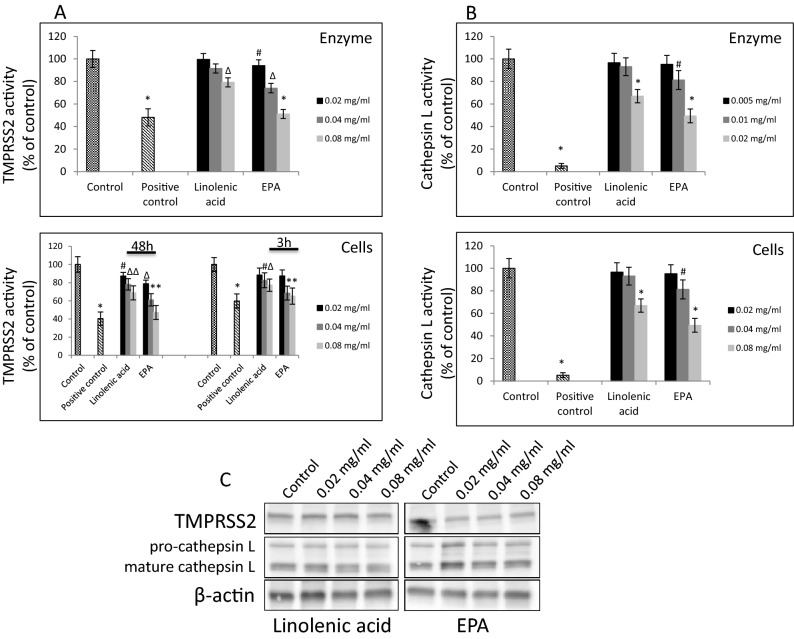
Effect of selected FAs on TMPRSS2 and Cathepsin L proteases. (**A**) Purified TMPRSS2 enzyme at 1 µM was incubated with selected FAs at different concentrations for 1 h at RT followed by addition of fluorogenic substrate at 10 µM concentration for 30 min. Fluorescence signal was measured at Ex/Em = 360/440 nm using spectrofluorimeter (upper panel). A549 cells were treated with indicated FAs at different concentrations for 3 h and 48 h at 37 °C and enzymatic activity was measured by addition of 0.2 mM fluorogenic substrate and incubation for 30 min. at 37 °C. Fluorescence signal was measured at Ex/Em = 360/440 nm using spectrofluorimeter (lower panel). Data are presented as % of control ± SD; #*p* ≤ 0.05, ∆*p* ≤ 0.01, *p ≤ 0.001. Control—0.025% DMSO, positive control—50–100 μM camostat mesylate. (**B**) Purified cathepsin L enzyme at 0.02 ng/µl was incubated with selected FAs at different concentrations for 1 h at RT followed by addition of fluorogenic substrate at 10 µM concentration for 30 min. Fluorescence signal was measured at Ex/Em = 360/440 nm using spectrofluorimeter (upper panel). A549 cells were treated with indicated lipids at different concentrations for 24 h at 37 °C and enzymatic activity was measured by addition of 0.2 mM fluorogenic substrate and incubation for 30 min. at 37 °C. Fluorescence signal was measured at Ex/Em = 360/535 nm using spectrofluorimeter (lower panel). Data are presented as % of control ± SD; #*p* ≤ 0.05, *p ≤ 0.001. Control—0.025% DMSO, positive control—0.1 μM E-64. (**C**) Western blot analysis of TMPRSS2 and cathepsin L expression in A549 cells treated with indicated FAs with different concentration for 48 h. Detection was done using rabbit anti-TMPRSS2 monoclonal antibody at 1:1000 and mouse anti-cathepsin L antibody at 1:200. Experiments were done in triplicate and repeated three times. Data are presented as % of lipid-free control ± SD.

Since, the SARS-CoV-2 enters hACE2 cells through endocytosis, and at the same time “priming” of spike protein is required, we utilized cell-free and cell-based assays to test the ability of linolenic acid and EPA to affect activity and expression of TMPRSS2 and cathepsin L enzymes that seem to be critical in the cellular binding and entry process. As presented in Fig. 6A linolenic acid used up to 0.08 mg/ml, statistically significantly inhibited enzymatic activity of recombinant TMPRSS2 protease by about 15%. The enzymatic activity of TMPRSS2 measured in A549/hACE2 cells was also decreased in the presence of linolenic acid by about 10–15%. At the same concentration, EPA showed to be more effective, significantly inhibiting enzymatic activity of recombinant TMPRSS2 protease by about 51%, and about 40–55% in A549/hACE2 cells. In both cases, the inhibitory effect was dose-dependent and consistent at concentrations showing, also, inhibition of cellular binding.

Next, we examined the ability of these PUFAs to inhibit cathepsin L activity, also shown to be relevant in SARS-CoV-2 entry and endosomal egress. Consistent results were obtained when assessment was performed with purified enzyme or with A549/hACE2 lysed cells after 24 h’ incubation, respectively. As shown in Fig. 6B, linolenic acid effectively inhibited enzymatic activity of recombinant cathepsin L by about 20%, and about 27% reduction was observed in A549/hACE2 cells. Similarly to the effects on TMPRSS2 protease, the EPA was more effective, and significantly inhibited enzymatic activity of recombinant cathepsin L by about 73%, and about 52% in A549/hACE2 cells. However, inhibitory effect on cathepsin L activity in cell lysates was achieved with concentrations four times higher than with these used in experiments utilizing recombinant enzyme. In both cases, the effect was dose-dependent. Also, Western blot analysis showed that neither linolenic acid nor EPA affected TMPRSS2 and cathepsin L expression at protein levels (Fig. 6C, and Supplementary Figure S1 and S2).

## Discussion

It was earlier reported that FAs, unsaturated fatty acids in particular, mediate antiviral activity through different mechanisms^27,31–38^. In general, their antimicrobial properties are concentrated on targeting microbial cell membranes, the generation of free radicals, and formation of cytotoxic lipid peroxides or bioactive immune-modulating metabolites. Free FAs such as oleic acid, arachidonic acid and linoleic acid have shown efficacy in inactivating enveloped viruses such as herpes, influenza, Sendai, and Sindbis^27,31^. Furthermore, exogenous supplementation of linoleic acid or arachidonic acid in infected cells, significantly suppressed replication of the HCoV-229E virus and the highly pathogenic Middle East Respiratory Syndrome coronavirus (MERS-CoV)^33^. Toelzer et al. in their 2.85 Å cryo-EM structure of SARS-CoV-2 spike glycoprotein revealed that the RBDs tightly bind the linoleic acid in three composite binding pockets and thus, by the stabilizing of a locked S conformation, can reduce interaction with the ACE2 receptor^30^. Additionally, Elfiky A. reported that his results showed moderate binding affinity for linolenic acid to the substrate-binding domain β (SBDβ) of the cell-surface Heat Shock Protein A5 (HSPA5), also named GRP78 or BiP, which was identified as yet another recognition site, beside ACE2, for the SARS-CoV-2 spike protein^36^.

Here, we provide evidence that PUFAs, predominantly linolenic acid, linoleic acid, and EPA inhibit attachment of pseudo-typed enveloped SARS-CoV-2 virions to the human ACE2 receptor through interacting directly with the RBD sequence. We also show that this attachment of spike-enveloped SARS-CoV-2 pseudo-virions is reduced particularly upon linolenic acid and EPA treatment, either when incubated directly with virions before adding to the cells, added to cells together with the virions, or added after cellular exposure to virions. Additionally, we observed that, while spike-expressing cell attachment and fusion to hACE2-expressing cells was affected by these PUFAs at non-toxic concentrations, they did not affect the activity of recombinant hACE2 or bond to this receptor directly. Since neither of the latter PUFAs affected the hACE2 receptor, even when applied at their highest concentrations; this would imply that linolenic acid and EPA do not directly interfere with the host cognate receptor, although they may be still acting by altering the properties of the cell membranes of the host, and hence ACE2 performance as an enzyme-receptor. On the other hand, because PUFAs are lipophilic molecules, they could interfere with the viral envelope itself, changing its dynamics and altering its receptor function. It was earlier demonstrated that PUFAs modify host membrane fluidity and at the same time inactivate viruses by disrupting their envelopes^27,31^. The changes in membrane fluidity attributed to decreased rigidity may distress the conformation of both the host and viral proteins and be determining for the SARS-CoV-2 virus interaction as well. All this reflects PUFAs’ characteristic as compounds that can penetrate cell surfaces. Their penetrating potential may rely on or be endowed with a cell-penetrating competence, similar to a group of proteins classified as cell-penetrating peptides (CPPs), since their structure is portrayed with a high degree of amphipathicity, where hydrophilic head and hydrophobic tail are distinct on the ends of the chain.

In order to gain a deeper insight as to how PUFAs inhibit viral entry, we looked at host membrane protease TMPRSS2 and endosomal protease cathepsin L, which have been shown to be critical in this process^20–25^. Linolenic acid and EPA inhibited the activity of these proteases in both cell-free and cell-based assay, but not their expression. Interestingly, EPA proved to be more effective than linolenic acid in inhibiting activity of these proteases. Also, EPA’s inhibitory effect on cathepsin L, which its putative function involves viral scission^24,25^, was more pronounced than on TMPRSS2. Interestingly, inhibitory effect on cathepsin L activity in cell-free experiment was attained with concentrations lower that with tese used in cell-based experiment. In this regard, inhibition of cathepsin L activity by EPA seems to be rather directed and specific. TMPRSS2 activity, on the other hand, since was interiorly affected than cathepsin L, could likely be allosterically affected by these FAs. Besides cell-penetration, FAs adopt an almost flat conformation or a spherical liposomal interface, which allows contact of hydroxyl groups with the aqueous environment acting via electrostatic forces as well. All this could interrupt the contact between the host membrane the viral envelope and subsequently inhibit SARS-CoV-2 attachment and entry upon FAs treatment.

In conclusion, we identified that FAs, PUFAs in particular, have an anti-SARS-CoV-2 efficacy. Predominantly, linolenic acid and EPA showed noticeable direct inhibitory effect on viral binding and also activity of host proteases TMPRSS2 and cathepsin L, rather than the ACE2 receptor. However, owing to FAs’ ability to incorporate into the lipid membranes, they may also destabilize both host and viral bilayers, consequently affecting their curvature and properties. Further studies are needed to unravel other mechanisms and to expand our understanding of FAs’ efficacy against SARS-CoV-2 infectivity.

## Material and methods

### Cell lines, constructs, and pseudo-viruses

Human alveolar epithelial cell line A549 was obtained from ATCC (American Type Culture Collection) (Manassas, VA). Human alveolar epithelial cell line A549, stably overexpressing hACE2 receptor, was obtained from GenScript (Piscataway, NJ). Both cell lines were maintained in Dulbecco’s MEM containing 10% fetal bovine serum, 100 U/ml penicillin, and 100 μg/ml streptomycin. Pseudo-virus particles with spike glycoprotein as the envelope protein with eGFP and luciferase (eGFP-luciferase-SARS-CoV-2 spike glycoprotein pseudo-typed particles) and pseudotyped ΔG-luciferase (G*ΔG-luciferase) rVSV were purchased from Kerafast (Boston, MA). Bald pseudo-virus particles with eGFP and luciferase (eGFP-luciferase-SARS-CoV-2 pseudo-typed particles) were purchased from BPS Bioscience (San Diego, CA). Lentiviral particles carrying human TMPRSS2 were from Addgene (Watertown, MA).

### Test compounds, antibodies and inhibitors

All lipid-soluble vitamins and FAs, except eicosapentaenoic acid, docosahexaenoic acid (DHA), and vitamin A (all-trans-retinol) that were purchased from Cayman Chemical Company (Ann Arbor, MI), were purchased from Sigma (St. Louis, MO). All inhibitors were from Cayman Chemical Company (Ann Arbor, MI). All antibodies were from R&D Systems (Minneapolis, MN) unless indicated otherwise.

### Binding and entry assays

#### SARS-CoV-2 RBD binding to hACE2

Binding/neutralization reaction was performed using a SARS-CoV-2 surrogate virus neutralization test kit that can detect either antibodies or inhibitors that block the interaction between the receptor-binding domain (RBD) of the SARS-CoV-2 spike protein with the hACE2 cell surface receptor (GenScript, Piscataway, NJ). For screening assay 2.5 mg/ml of tested lipids and lipid-soluble vitamins were incubated with either HRP-conjugated receptor-binding domain (RBD fragment) of SARS-CoV-2 spike S1 domain, or with human ACE2 receptor (hACE2) immobilized on a 96-well plate for 30 min at 37 °C. Next, samples that were incubated with RBD fragment were transferred into a 96-well plate with immobilized hACE2 receptor and incubated for an additional 15 min at 37 °C, whereas hACE2-immobilized plates already incubated with different lipids were washed four times with washing buffer and treated with HRP-conjugated RBD fragments and incubated for 15 min at 37 °C. Next, plates were washed four times with washing buffer and developed with TMB substrate solution for up to 5 min, followed by the addition of stop buffer. Optical density was immediately measured at 450 nm with a plate reader (Molecular Devices, San Jose, CA). Positive and negative controls were provided by the manufacturer. Results are expressed as a percentage of experimental lipid-free control (mean + /- SD, n = 5).

#### SARS-CoV-2 pseudo-virus binding to hACE2

Binding/neutralization reaction was performed using the general GenScript-developed protocol and recommendation with little applied adjustments. Briefly, the eGFP-luciferase-SARS-CoV-2 spike S1 pseudo-virus was either pre-incubated at 37 °C with selected FAs (i.e., linolenic acid and eicosapentaenoic acid) at concentrations ranging from 0–2.5 mg/ml (linolenic acid: 0–9.0 mM, EPA: 0–8.3 mM) for 1 h, before being added into a plate with human A549 lung epithelial cells overexpressing hACE2, simultaneously with the selected FAs, or was added into the plate and the 1-h post-exposure followed treatment with selected FAs. Samples were incubated for an additional 1 h, 3 h, and 48 h (in 48 h experiment the eGFP-luciferase-CoV-2 spike S1 pseudo-virus was spin-inoculated at 1200× g for 45 min or not), at 37 °C. After the incubation period, the plates were washed three times with washing buffer (provided by the manufacturer) and either HRP-signal (primary anti-SARS-CoV-2 spike protein antibody at 1:1000 followed by HRP-conjugated secondary antibody at 1:2500) were used in standard enzyme-linked immunosorbent assay (i.e., 1-h and 3-h experiments), or the transduction efficiency was measured by quantification of the luciferase activity using a Luciferase Glo kit (Promega, Madison, WI) (i.e., 48-h experiments with and without spinfection) and a plate reader (Molecular Devices, San Jose, CA). In 1-h and 3-h experiments, positive and negative controls were the same as those used in SARS-CoV-2 RBD binding to hACE2 assay and were provided by the manufacture. In 48-h experiments, the positive control was bald eGFP-luciferase-SARS-CoV-2 pseudo-typed particles, and the negative control was ΔG-luciferase rVSV pseudo-typed particles. Results are expressed as a percentage of experimental lipid-free control (mean + / SD, n = 5).

#### SARS-CoV-2 spike protein expressing cells binding to soluble hACE2

To transduce cells with eGFP-luciferase-SARS-CoV-2 spike S1 lentivirus vector (GenScript, Piscataway, NJ), A549 cells seeded into a 6-well plate in the presence of complete growth medium were treated with 8 µl/ml polybrene (Sigma, St. Louis, MO) for 30 min, followed by the addition of eGFP-luciferase- CoV-2 spike S1 lentivirus at MOI = 40 (our previous preliminary results showed an almost 100% transduction rate can be achieved with this MOI), and spin-inoculation at 800× g for 1.5 h. After 24 h at 37 °C incubation, cells were fed with fresh complete growth medium. After 48 h post-inoculation, cells were detached with 1 mM EDTA, washed twice with 1 × PBS (phosphate-buffered saline) supplemented with 3% FBS (fetal bovine serum), and treated with indicated concentrations of FAs for 1 h, followed by incubation with 5 µg/ml of soluble hACE2 (Sigma, St. Louis, MO) for 1 h on ice^8^. After washing three times with 3% FBS in 1 × PBS, cells were transferred into plates immobilized with human monoclonal anti-ACE2 antibody at 10 µg/ml (Cayman Chemical Company, Ann Arbor, MI). After 1 h’s incubation, wells were washed three times with 3% FBS in 1 × PBS, and the fluorescence signal was measured at Em/Ex 488/535 nm wavelength with a plate reader (Tecan Group Ltd., Switzerland). Positive and negative controls were the same as those used in SARS-CoV-2 RBD binding to hACE2 assay, and were provided by the manufacturer. Results are expressed as a percentage of experimental lipid-free control (mean + /− SD, n = 5).

### Cell–cell fusion assay

Fusion assay was performed according to a previously published report^8^. Briefly, A549 cells transduced with eGFP-luciferase-SARS-CoV-2 spike S1 lentivirus vector (GenScript, Piscataway, NJ) were detached with 1 mM EDTA, treated with selected FAs at 20–160 µg/ml (linolenic acid: 71.8–574.7 mM, EPA: 66.1–529.1 mM) concentrations for 1 h at 37 °C and overlaid on 95% confluent human A567 lung epithelial cells overexpressing hACE2. After 4 h’ incubation at 37 °C, images of syncytia were captured with a Zeiss Axio Observer A1 fluorescence microscope (Carl Zeiss Meditec, Inc, Dublin, CA). All experiments were done in triplicate and repeated three times. The positive control was 20 μg/ml anti-ACE2 antibody. Results are expressed as a percentage of experimental lipid-free control (mean + /− SD, n = 3).

### TMPRSS2 activity assay

TMPRSS2 activity assay on cells was performed according to a previously published report^39^. To determine the TMPRSS2 activity on cells, A549 cells with overexpressed TMPRSS2 were seeded in 48-well plates. Forty-eight hours or 3 h prior to the protease activity measurements, the cells were treated with selected FAs at 20–80 µg/ml (linolenic acid: 71.8–287.3 mM, EPA: 66.1–264.6 mM) concentrations. Next, cells were washed with phenol red-free DMEM, and the protease activity was assessed by incubation of cells with the 0.2 mM fluorogenic substrate Mes-D-Arg-Pro-Arg-AMC in 50 mM PBS (pH = 7.4) for 30 min at 37 °C (Fisher Scientific, Pittsburgh, PA). Hydrolysis of the peptide was monitored by the measurement of fluorescence intensity using a Tecan fluorescence spectrometer at Em/Ex = 360/440 nm (Tecan Group Ltd., Switzerland). The positive control was 50 μM camostat mesylate. Results are expressed as a percentage of experimental lipid-free control (mean + /− SD, n = 5).

To determine the inhibitory effect of selected FAs on activity of recombinant TMPRSS2 protein, 10 µM fluorogenic peptide Boc-Gln-Ala-Arg-AMC was added to linolenic acid or EPA diluted at 20–80 µg/ml concentrations. To this reaction 1 µM of TMPRSS2 enzyme (Creative BioMart, Shirley, NY) in assay buffer (50 mM Tris pH = 8, 150 mM NaCl) was added. Following 1 h’s incubation at RT, detection of the fluorescent signal was done using a Tecan fluorescence spectrometer at Em/Ex = 360/440 nm (Tecan Group Ltd., Switzerland). The positive control was 100 μM camostat mesylate. Results are expressed as a percentage of experimental lipid-free control (mean + /− SD, n = 5).

### Cathepsin L activity assay

Cathepsin L activity assays in cells were performed utilizing a Cathepsin L Activity Assay kit (Abcam, Cambridge, MA) according to the manufacturer’s protocol. Briefly, A549 cells were seeded in 6-well plates and allowed to adhere for 24 h or until reaching 90–95% of confluency. Next, cells were treated with selected FAs at 20–80 µg/ml (linolenic acid: 71.8–287.3 mM, EPA: 66.1–264.6 mM) concentrations for an additional 24 h, washed with cold 1 × PBS, and lysed with 100 μl of chilled CL Buffer on ice for 10 min. Next, samples were centrifuged for 2 min at 4 °C to remove any insoluble material. Supernatants were collected and transferred to clean tubes that were kept on ice. Next, enzymatic reaction was set up by mixing treated sample wells containing 50 μl sample, untreated sample wells (control) containing 50 μl sample, background control wells containing 50 μl sample, positive control containing 5 μl reconstituted positive control in 45 μl CL buffer, and negative control containing 5 μl reconstituted positive control in 45 μl CL buffer and 2 μl CL inhibitor. Next, 50 μl CL Buffer and 1 μl 1 mM DTT was added to each well. Finally, 2 μl 10 mM CL substrate Ac-FR-AFC (0.2 mM final concentration) was added to each well, except to the background control wells. Next, plates were incubated at 37 °C for 1 h and the fluorescence signal was measured at Ex/Em = 360/535 nm with a microplate reader (Tecan Group Ltd., Switzerland). Results are expressed as a percentage of experimental lipid-free control (mean + /− SD, n = 5).

To determine the inhibitory effect of selected FAs at 5–20 µg/ml (linolenic acid: 17.95–71.83 mM, EPA: 16.53–66.15 mM) concentrations on the activity of recombinant cathepsin L protein, a Cathepsin L Activity Screening Assay kit (BPS Bioscience, San Diego, CA) was utilized and run according to the manufacturer’s protocol. Briefly, to cathepsin L enzyme (0.02 ng/μl) selected FAs were added and the reaction mix was incubated for 15 min at RT. The positive control was the sample containing only cathepsin L enzyme, and the negative control was a sample containing cathepsin L enzyme and cathepsin L enzyme inhibitor E-64 (0.1 μM). Next, cathepsin L fluorogenic substrate (Ac-FR-AFC) (10 μM) was added to each well, and the plate was incubated for 1 h at RT, protected from light. The fluorescence was measured at Ex/Em = 360/440 nm using a microplate reader (Tecan Group Ltd., Switzerland). Results are expressed as a percentage of experimental lipid-free control (mean + /− SD, n = 5).

### ACE2 activity assay

To determine the inhibitory effect of selected FAs on the activity of recombinant ACE2 protein, an ACE2 Activity Screening Assay kit (BPS Bioscience, San Diego, CA) was utilized and run according to the manufacturer’s protocol. Briefly, selected FAs at 20–80 μg/ml (linolenic acid: 71.8–287.3 mM, EPA: 66.1–264.6 mM) concentrations were added to hACE2 enzyme (0.1 ng/μl) and the reaction mix was incubated for 15 min at RT. The positive control was the sample containing only ACE2 enzyme, and the negative control was a sample containing ACE2 enzyme and 10% DMSO. Next, ACE2 fluorogenic substrate (10 μM) was added to each well, and the plate was incubated for 1 h at RT, protected from light. The fluorescence was measured at Ex/Em = 535/595 nm using a microplate reader (Tecan Group Ltd., Switzerland). Results are expressed as a percentage of experimental lipid-free control (mean + /− SD, n = 5).

### ACE2 binding assay

To determine the inhibitory effect of selected FAs on binding to ACE2 receptor, an ACE2 Inhibitor Screening Assay kit (BPS Bioscience, San Diego, CA) was utilized and run according to the manufacturer’s protocol. Briefly, selected FAs at 20–80 μg/ml (linolenic acid: 71.8–287.3 mM, EPA: 66.1–264.6 mM) concentrations were added to ACE2 receptors immobilized on the plate (1.0 μg/ml), and the reaction mix was incubated for 1 h at RT. The positive control was 50% DMSO. Next, the plate was washed three times with washing buffer, blocked with blocking buffer for 1 h, and incubated with anti-ACE2 antibody at 1:500 dilution for 1 h at RT, followed by three times washing, blocking with blocking buffer, and incubation with HRP-conjugated secondary antibody at 1:1000 dilution for an additional 1hour at RT. The plates were again washed three times with washing buffer, and chemiluminescence signal was measured using ECL substrate A and ECL substrate B mixed 1:1 using a microplate reader (Tecan Group Ltd., Switzerland). All experiments were done in triplicate and repeated three times. Results are expressed as a percentage of experimental lipid-free control.

### Viability assay

MTT assay was used to assess cell viability. Briefly, A549 cells were seeded into a 96-well plate at a cell density of 4 × 10^4^ per well and allowed to adhere for 24 h, followed by treatment with serially diluted selected PUFAs for up to 48 h. Next, complete growth medium was replaced with a fresh one substituted with 5 mg/ml MTT, followed by incubation for 3 h at 37 °C. After removing the culture medium, 100 μl of methanol was added and the absorbance was measured at 570 nm using a microplate spectrophotometer (Molecular Devices, San Jose, CA). Results are expressed as a percentage of experimental lipid-free control (mean + /− SD, n = 8).

### Western blot analysis

A549 cells were treated with indicated concentrations of selected PUFAs and lysed using lysis buffer [50 mM Tris–HCl (pH = 7.4), 1% TritonX-100, 150 mM NaCl, 1 mM EDTA, 2 mM Na3VO4, and 1X Complete protease inhibitors (Roche Applied Science, Indianapolis, IN)]. The protein concentration was measured by the Dc protein assay (Bio-Rad, Hercules, CA). A 50 µg/well of protein was separated on 8–16% gradient SDS-PAGE gels (i.e., Tris-based electrophoresis using standard Laemmle's method) and transferred to a PVDF membrane. Proteins were detected either with commercially available anti-TMPRSS2 monoclonal antibody at 1:1000 dilution (Abcam, Cambridge, MA) or anti-cathepsin L monoclonal antibody at 1:200 dilution (Santa Cruz Biotechnology, Santa Cruz, CA), and anti-β-actin antibody at 1:1000 dilution as a loading control (Cell Signaling, Danvers, MA). WB images were acquired using the Azure cSeries system (Azure Biosystems, CA) and auto-exposure settings.

## Supplementary Information

Supplementary information.
