# Supplementary material for: Polyunsaturated ω-3 fatty acids inhibit ACE2-controlled SARS-CoV-2 binding and cellular entry
Source: Sci Rep. 2021 Mar 4;11:5207. doi: 10.1038/s41598-021-84850-1 (PMC7933164; doi:10.1038/s41598-021-84850-1)
Supplement: Supplementary file 1 — Supplementary information. [file 41598_2021_84850_MOESM1_ESM.pdf]

Supplementary Information

Polyunsaturated ω-3 fatty acids inhibit ACE2-controlled SARS-CoV-2 binding and cellular entry

Anna Goc[1*], Aleksandra Niedzwiecki[1*], Matthias Rath[1]
[1]Dr. Rath Research Institute, 5941 Optical Ct., San Jose, CA 95138, USA

Supplementary Figure S1

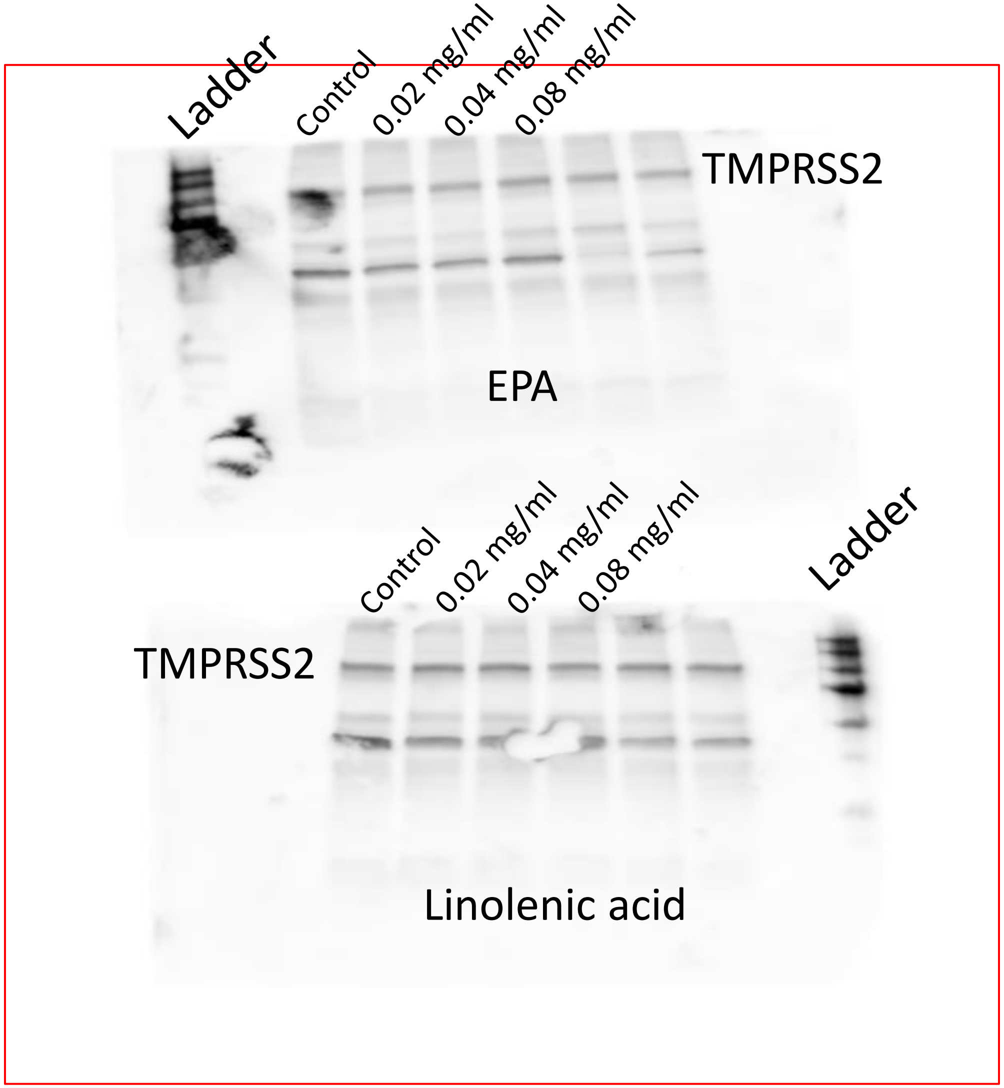

Supplementary Figure S1. Analysis of TMPRSS2 expression at protein level in A549 cells treated with indicated FAs with different concentration for 48h. Experiment was done using Western blot method with rabbit anti-TMPRSS2 monoclonal antibody at 1:1000 dilution. A 50 µg/well of protein was separated on 8-16% gradient SDS-PAGE gels. Original WB images were acquired using the Azure cSeries digital system and auto-exposure settings.

Supplementary Figure S2

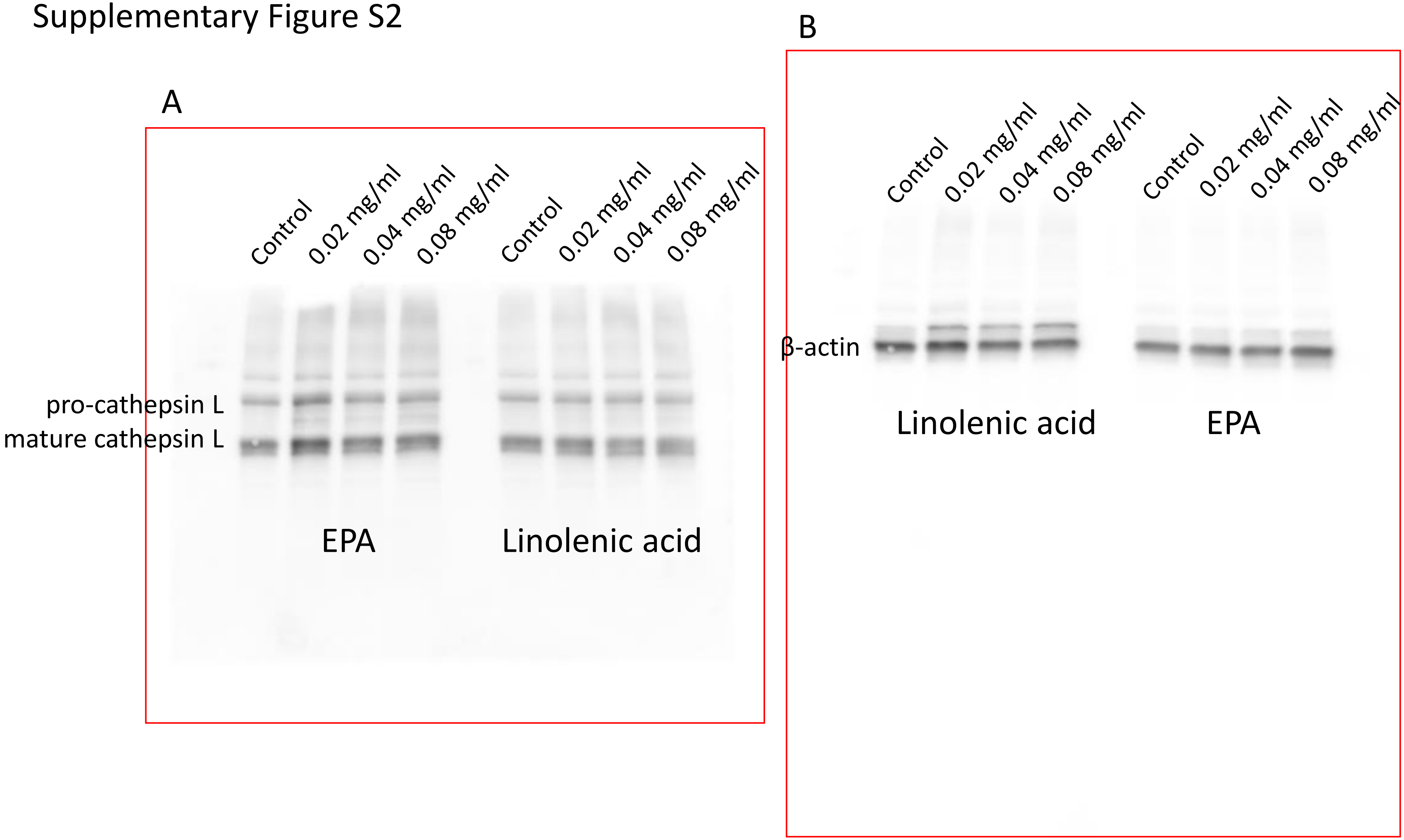

Supplementary Figure S2. Analysis of cathepsin L expression at protein level in A549 cells treated with indicated FAs with different concentration for 48h. Experiment was done using Western blot method with mouse anti-cathepsin L monoclonal antibody at 1:200 dilution (**A**) and anti-β-actin antibody at 1:1000 dilution as a loading control (**B**). A 50 µg/well of protein was separated on 8-16% gradient SDS-PAGE gels. Original WB images were acquired using the Azure cSeries digital system and auto-exposure settings.
